# The relationship between rice consumption and glioma: a case–control study in adults

**DOI:** 10.1038/s41598-021-85562-2

**Published:** 2021-03-16

**Authors:** Maryam Aghababaie Shahrestani, Parvane Saneei, Mehdi Shayanfar, Minoo Mohammad-Shirazi, Giuve Sharifi, Omid Sadeghi, Ahmad Esmaillzadeh

**Affiliations:** 1 Department of Community Nutrition, School of Nutrition and Food Science, Isfahan University of Medical Sciences, Isfahan, Iran; 2 Department of Clinical Nutrition and Dietetics, National Nutrition and Food Technology Research Institute, Shahid Beheshti University of Medical Sciences, Tehran, Iran; 3 Department of Community Nutrition, School of Nutritional Sciences and Dietetics, Faculty Member, Tehran University of Medical Sciences, P.O. Box 14155-6117, Tehran, Iran; 4 Endocrinology and Metabolism Research Center, Endocrinology and Metabolism Clinical Sciences Institute, Tehran University of Medical Sciences, Tehran, Iran; 5 Department of Community Nutrition, School of Nutrition and Food Science, Food Security Research Center, University of Medical Sciences, Isfahan, Iran; 6 Department of Education Development in Nutrition, School of Nutritional Sciences and Dietetics, Faculty Member, Tehran University of Medical Sciences, P.O. Box 14155-6117, Tehran, Iran

**Keywords:** Head and neck cancer, Nutrition, Neurological disorders

## Abstract

Previous studies have shown the effect of refined grains on various cancers; however, data on the link between rice consumption and brain cancer are scarce. We aimed to investigate the relationship between rice consumption and glioma in Iranian adults. Current hospital-based case–control study was done in Tehran between 2009 and 2011. Cases were individuals with pathologically confirmed glioma in a maximally 1 month of the disease diagnosis (n = 128). Controls were individuals, aged between 20 and 75 years, who were hospitalized or were outpatients referred to other wards of the same hospital (n = 256). Cases and controls were frequently matched in terms of age and gender. Usual dietary intakes of participants, including rice consumption, during the preceding year were examined using a Block-format validated semi-quantitative 126-item food frequency questionnaire. Compared with participants in the lowest tertile of rice consumption (< 181 g/day), those in the highest tertile (≥ 279 g/day) had 2.47 times greater chance for having glioma (OR: 2.47, 95% CI 1.44–4.23). This relationship was also seen when potential confounders including demographic variables, energy and dietary intakes as well as body mass index were taking into account; such that individuals in the top tertile of rice consumption had 2.46 times greater odds of glioma compared with those in the bottom tertile (OR: 2.46, 95% CI 1.01–5.97). We found that rice consumption was positively associated with risk of glioma in adults. Further prospective studies are required to confirm this finding.

## Introduction

Glioma represents the most common type of adult brain tumor^1^. Glioma makes up about 31% of all central nervous system (CNS) tumors and 81% of all malignant brain tumors^2^. The incidence of primary malignant CNS tumors ranges from 2.1 to 5.8 per 100,000 people in the world^3^. In Iran, it has been estimated that 2.73 per 100,000 people are affected by the disease^4^. This tumor is associated with significant mortality and morbidity; the estimated 5- and 10-year relative survival rates for all malignant brain and CNS tumors are 34.4% and 28.8%, respectively^5^. This malignant brain tumor could trigger seizures, hypercoagulation, venous thromboembolism, and mood and cognitive disorders^6^. In 2016, the disability-adjusted life years (DALYs) of brain and CNS cancers were 0.65% of total DALYs among Iranians^7^.

Little is known with regard to the role of dietary factors in the incidence of some cancers including brain tumors. Some investigations have showed inverse associations between whole grains intake and risk of breast and pancreatic cancers as well as cancer mortality^8–10^. On the other hand, consumption of refined grains has been examined in relation to the risk of colorectal, prostate, oral cavity, pharynx, esophagus and larynx cancers^11–13^. With regard to rice consumption, some researches investigated the relation with different cancers and reported no significant associations^14,15^, while some others have found significant positive associations with risk of upper aerodigestive tract and pancreatic cancers^16,17^. Data on the link between rice consumption and brain cancers are scarce. However, consumption of processed meat^18^ and sugar^19^ has been associated with elevated risk of brain cancers. In contrast, high intake of fruits, vegetables^20^ and fresh fish^21^ has been linked with a lower risk. In case of glioma, some studies found that vitamins in food or supplements such as vitamin E, A and C, some antioxidant agents and dietary patterns (such as Mediterranean dietary pattern) have protective effect against the disease^22–24^.

White rice is the major staple food of more than half of the world’s population, in particular those living in Asian countries. Studying the relation between rice intake and cancers is particularly relevant for Iranian population, where rice consumption contributes greatly to total energy intake. Iranians get more than 57 percent of their daily energy intake from carbohydrates, almost entirely from refined sources^25^. Also, Iran is the 13th biggest white rice consumer worldwide with an average annual per capita consumption of 34 kg^26^. Intake of white rice could stimulate insulin and insulin-like growth factor-1 (IGF-1) secretion and postprandial glycemia which implicate in the etiology of some cancers^27,28^. Both insulin and IGF-1 could act as growth factors for tumor cells. Although IGF-I is important in normal brain development^29^, it was found to be over-expressed in glioma^30^. Given the high mortality rate of brain cancer along with the high consumption of rice in the region, this study aimed to investigate the relationship between rice consumption and glioma in Iranian adult population. We hypothesized that consuming more rice might be associated with a higher risk of glioma.

## Methods and materials

### Participants

This study was a hospital-based case–control study conducted in Tehran between November 2009 and September 2011 (the detailed report on methods and data collection, have been previously published^22,24,31,32^. Briefly, the sample size of our study was calculated based on previous published studies that showed approximately 60% of the Iranian adults consume less fruits and vegetables than recommended. We assumed that low fruit and vegetable intakes would double the risk of glioma. With a power 80%, type I error of 0.05, and desired confidence interval of 0.95, the minimum required sample size was calculated to be 115 cases and 230 control subjects. So, we recruited 128 cases and 256 controls from the hospitals. The participation rate was 100% among cases and 89% among controls. Both cases and controls were selected by using a convenience-sampling method and based on inclusion criteria. Cases were adult individuals (with the age of 20–75 years old) with pathologically confirmed glioma (ICD-O-2 morphology codes 9380–9481) in a maximally 1 month of the disease confirmation and had been referred to the Department of Neurosurgery of the hospitals affiliated to Shahid Beheshti University of Medical Sciences. Individuals with a history of any type of other pathologically confirmed cancers, and those with a history of chemotherapy or radiotherapy (due to cancer) were not included in this investigation. Control group were adult individuals aged between 20 and 75 years old who were hospitalized or were outpatients who referred to other wards of the same hospital, preferably for reconstructive surgery or orthopedic problems. Individuals with any type of cancer, gastrointestinal disorder, liver disease, and metabolic or immune system dysfunction were not included in the study. In addition, being on a vegetarian or weight loss diet were other exclusion criteria. Cases and controls were frequently matched in terms of age (± 5) and gender. All cases and controls provided informed written consent. All methods were performed in accordance with the relevant guidelines and regulations. The present study was ethically approved by the Medical Ethics Committee of the Isfahan University of Medical Sciences, Isfahan, Iran.

### Assessment of rice consumption

Usual dietary intakes of participants during the preceding year (to the diagnosis of glioma in cases and the interview in controls) were examined by trained interviewers, using a food frequency questionnaire (FFQ)^33^. Participants were requested to report their usual intake of food items considering the given portion size on a daily, weekly or monthly basis. The applied Block-format validated semi-quantitative FFQ consisted of a list of 126 food items with a standard serving size for each food^33^. Nutritionists, who have received the required training on the anthropometric measurement techniques, how to fill out food frequency questionnaire and physical activity questionnaire, and how to collect these data, interviewed the participants. The interviewers were unaware of the research hypotheses, but he/she was aware of the participants’ condition (in terms of having a disease). The reported values for each food were converted into grams using household measures^34^. Interviews were carried out in the presence of individuals involved in provision and cooking of food. Completed FFQs were analyzed using Nutritionist IV (N4) software (First Databank, San Bruno, CA, USA). Rice consumption was calculated by summing up rice from all foods in the questionnaire.

Validation study of this FFQ revealed good correlation between dietary intakes assessed by FFQ and those obtained from 24 dietary recalls (two 24-h recalls per month)^33^. The energy adjusted correlation coefficients between the dietary intakes obtained from the FFQ and those from the multiple 24-h dietary recalls were 0.65, 0.68 and 0.65 for vitamin E, β-carotene and vitamin C. Also, the reliability of the FFQ was assessed by comparing nutrient intakes obtained from two questionnaires that filled within a year. The correlation coefficients for the reliability of the FFQ for dietary vitamin E, β-carotene and vitamin C were 0.78, 0.84 and 0.83. The correlation coefficients comparing carbohydrate intake based on the FFQ and 24-h diet recalls were 0.75^33^. Overall, these data indicated that the FFQ provided reasonably valid measures of the average long-time dietary intakes.

### Assessment of glioma

Glioma was diagnosed based on pathological test by using International Classification of Diseases for Oncology second edition (ICD-O-2) and morphology codes 9380–9481. Only patients with a maximum 1 month of the confirmation of glioma were included in the study.

### Assessment of other variables

Participant's weight was measured by a nutritionist using a digital scale to the nearest 0.5 kg, while the subjects were wearing the least clothing and no shoes. Height was measured in a standing position with a tape measure to the nearest 1 cm with the shoulders in a normal position. Body mass index (BMI) was calculated as weight (kg) divided by height (in meters squared). Participants’ physical activity during the last year was assessed using International Physical Activity Questionnaire (IPAQ) and expressed as Metabolic Equivalent Task-hours per week (MET-h/wk). Required information about age, sex, marital status, education, high risk occupation, residential area, duration of cell phone use, supplement use, family history of cancer and glioma, history of allergy or head trauma, history of hypertension, history of exposure to the radiographic x-ray, exposure to chemical materials within 10 years, drug use, personal hair dye use and frequent use of barbecue, canned foods or microwave was evaluated using a pre-tested questionnaire. Based on the previous studies, being formers living near electromagnetism area and cell phone antenna in the last 10 years, consuming fried food at least twice per week, using barbecue or microwave and consumption of canned foods were considered as covariates.

### Statistical methods

First, we categorized individuals in tertiles of rice intake (T1: < 181 g/day, T2: 181 to < 279 g/day, T3: ≥ 279 g/day), in order to assess the relationship between rice consumption and glioma. General characteristics and dietary intakes of cases and controls were assessed using independent samples Student’s t test. General characteristics of study participants across tertiles of the rice consumption were compared using one-way ANOVA for continuous variables and χ2 test for categorical variables. Association of the rice consumption with glioma was assessed using logistic regression in different models. The covariates were selected based on previous investigations^35–37^. First, we controlled for main confounders, including age (continuous), sex (male/female) and energy intake (kcal/day). Further adjustments were made for other modifiable and unmodifiable risk factors including physical activity (continuous), family history of cancer (yes/no), family history of glioma (yes/no), marital status (married/single/divorced), education (university graduated/non university graduated), high risk occupation (farmer/ non-farmer), high risk residential area (yes/no), duration of cell phone use (continuous), supplement use (yes/no), history of exposure to the radiographic x-ray (yes/no), history of head trauma (yes/no), history of allergy (yes/no), history of hypertension (yes/no), smoking (smoker/non-smoker), exposure to chemicals (yes/no), drug use (yes/no), personal hair dye use, frequent fried food intake (yes/no), frequent use of barbecue, canned foods or microwave (yes/no) in the second model. In the third model further adjustment were made for dietary intakes including whole grains, red and processed meats, white meats, fruits, vegetables, legumes and nuts, and dietary fat, refined grain (except for rice) and caffeine. Additional controlling was performed for BMI in the last model, to obtain an independent relation from obesity. In all models, the first tertile of rice consumption was considered as the reference category. The stability of the models was considered to be disturbed by the multicolinearity if tolerance was under 0.1. Tolerance is a statistic applied to examine how much the independent variables are linearly related to one another. It is calculated as 1 − R^2^ for an independent variable, when it is predicted by the other independent variables already included in the analyses. Goodness-of-fit for the models was tested using the Hosmer–Lemeshow test. All the statistical analyses were carried out using SPSS (SPSS Inc., version 18). *P* values were considered significant at < 0.05.

## Results

General characteristics of the study participants in across case and control groups and categories of rice consumption are reported in Table 1. Individuals with glioma were more likely to have high-risk jobs (*P* = 0.003), have a family history of brain tumors (*P* < 0.001) and be frequently exposed to chemicals and radiographic X-ray (*P* = 0.01) in comparison to healthy individuals. The prevalence of head trauma and smoking was higher in cases than controls (*P* = 0.004 and *P* = 0.003, respectively). Cases were less likely to use hair dye in the last 10 years than controls (*P* < 0.001). Age and BMI distribution was not significantly different in cases and controls (*P* = 0.65 and *P* = 0.75, respectively). Other variables (including sex (*P* = 0.99), education (*P* = 0.22), family history of cancer (*P* = 0.90), supplement use (*P* = 0.36), history of allergy (*P* = 0.40) and hypertension (*P* = 0.28)) were not significantly different between case and control groups. Participants in the highest tertile of rice consumption had higher BMI (*P* = 0.01), were more likely to be younger (*P* < 0.001), male (*P* < 0.001), at high-risk areas (*P* < 0.001), frequent medication users (*P* = 0.02), frequent barbecue users (*P* < 0.001), and were highly exposed to chemicals (*P* = 0.01) compared with those in the lowest tertile. Compared with those in the bottom tertile, individuals in the top tertile of rice consumption were less likely to use hair dye in the last 10 years (*P* = 0.002). No other significant differences were found across tertiles of rice consumption.

**Table 1:** General characteristics of study participants across tertiles of rice consumption.

	Groups		*P*^a^	Tertiles of rice consumption			
	Cases (n = 128)	Controls (n = 256)		T_1_ (< 181 g/day) (n = 129)	T_2_ (181 to < 279 g/day) (n = 121)	T_3_ (≥ 279 g/day) (n = 134)	*P*^a^
Age (y)	43.4 ± 14.6	42.7 ± 13.3	0.65	45.23 ± 13.69	44.99 ± 14.62	39.03 ± 12.14	< 0.001
Females (%)	41	42	0.99	54.3	49.6	22.4	< 0.001
Body mass index (kg/m^2^)	26.2 ± 4.2	26.1 ± 3.8	0.75	25.45 ± 3.62	26.16 ± 3.98	26.88 ± 4.26	0.01
Married (%)	79	80	0.66	78.3	77.7	82.8	0.28
University graduated (%)	12	17	0.22	18.6	17.4	9.7	0.09
High-risk jobs^b^ (%)	10	3	0.003	3.1	4.1	8.2	0.14
High-risk residential area^c^	30	21	0.05	17.1	19	36.6	< 0.001
Duration of cell phone use (y)	2.85 ± 2.8	3.70 ± 2.56	0.003	3.46 ± 2.85	3.16 ± 2.69	3.62 ± 2.57	0.40
Exposure to x-ray (%)	16	7.4	0.01	11.6	6.6	11.9	0.29
History of head trauma (%)	44	29	0.004	31.8	30.6	38.8	0.32
Allergy (%)	25	29	0.40	31.8	27.3	24.6	0.43
Hypertension (%)	2	5	0.28	1.6	7.4	3.7	0.06
Current smoker (%)	16	25	0.003	20.2	18.2	26.9	0.21
Frequent fried food intake^d^ (%)	91	78	0.001	80.6	80.2	85.8	0.41
Frequent use of barbecue^e^ (%)	16	12	0.21	7.8	8.3	23.1	< 0.001
Frequent microwave use^e^ (%)	8	19	0.002	15.5	14	16.4	0.87
Frequent canned foods intake^e^ (%)	6	7	0.52	6.2	7.4	4.5	0.60
Drug usage (%)	8	5	0.36	2.3	5	10.4	0.02
Personal hair dye use (%)	22	41	< 0.001	45.7	33.9	24.6	0.002
Exposure to chemicals (%)	20	11	0.01	7.8	12.4	20.1	0.01
Family history of glioma (%)	19	5	< 0.001	6.2	14	10.4	0.12
Family history of cancer (%)	33	34	0.90	31.8	30.6	38.1	0.39
Supplement use (%)	8	16	0.36	19.4	9.1	10.4	0.30
Physical activity (Met-h/wk)	34.7 ± 6.3	33.8 ± 5.5	0.12	33.86 ± 5.27	33.79 ± 5.36	34.75 ± 6.6	0.33

Data are presented as mean ± SD or percent.

^a^Obtained from one-way ANOVA, independent samples Student’s t test or chi-square test, were appropriate.

^b^Farmers were considered as having a high-risk occupation.

^c^Individuals who lived in places near to the electromagnetic fields, cell phone and broadcast antennas in the last 10 years were defined as living in high-risk areas.

^d^Individuals with at least two times of fried food intake per week were considered as frequent fried food users.

^e^Those with at least two times per week of barbeque use, microwave use as well as consumption of canned foods were considered as frequent users.

**Figure 1:**
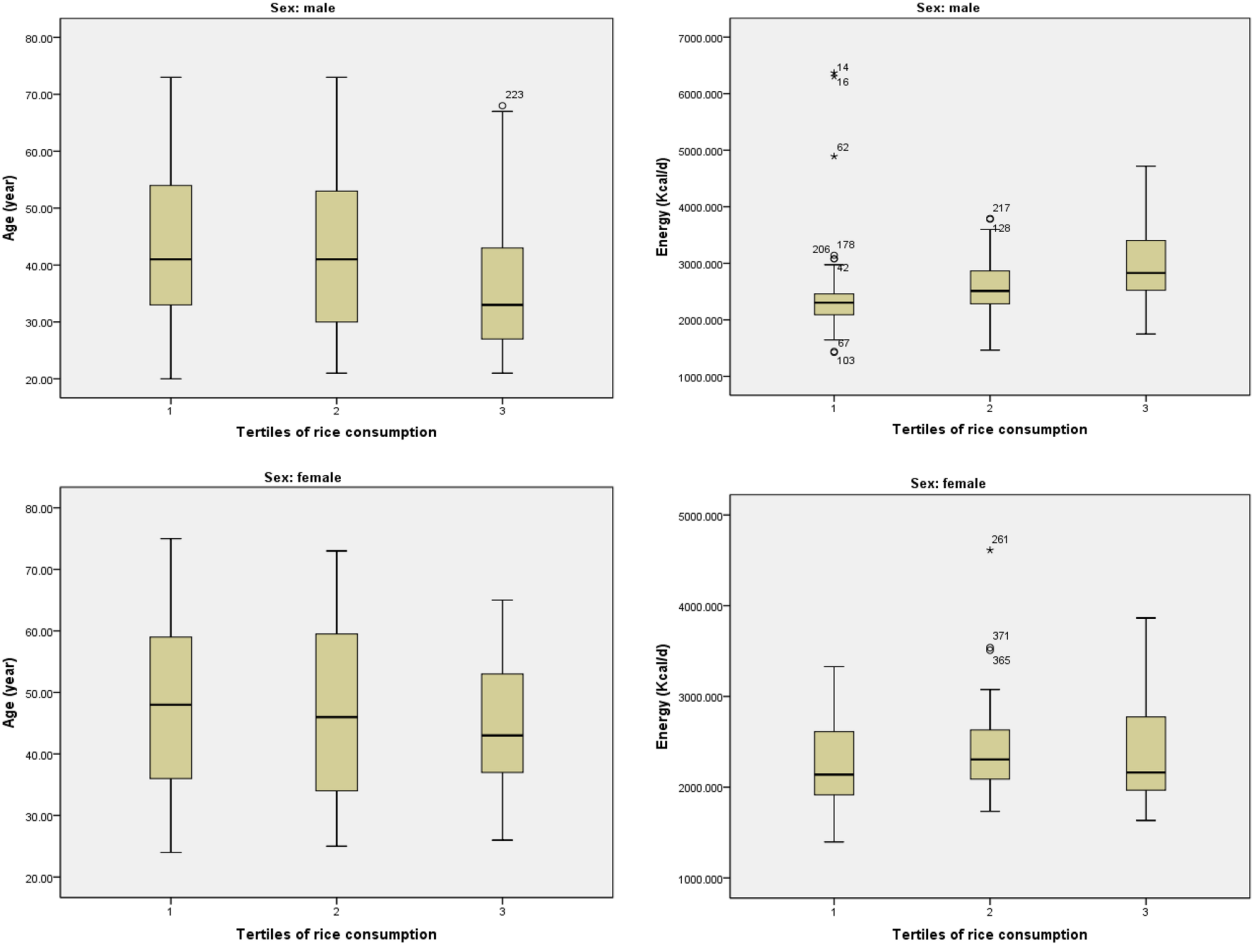
Distribution of age and energy intake of men and women across tertiles of rice consumption.

Distribution of age and energy intake of men and women across tertiles of rice consumption is depicted in Fig. 1. Male participants in the highest tertile of rice consumption had a relatively younger average age and a higher daily energy intake. There was no significant difference in case of age and energy intake in categories of rice consumption in female participants.

Dietary intakes of participants across tertiles of rice consumption are provided in Table 2. Compared with controls, cases had higher intakes of sodium, refined grains (*P* < 0.001), red and processed meats (*P* = 0.03) and partially hydrogenated vegetable oils (*P* < 0.001) and lower intakes of fats (*P* = 0.05), calcium (*P* = 0.001), selenium (*P* = 0.02), vitamin E (*P* = 0.03), whole-grains (*P* = 0.03), fruits (*P* = 0.005), vegetables (*P* = 0.07), dairy products (*P* = 0.001), legumes and nuts (*P* = 0.01), and non-hydrogenated vegetable oils (*P* = 0.03). Participants in the third tertile of rice consumption had high intakes of energy (*P* < 0.001), protein (*P* < 0.001), carbohydrate (*P* < 0.001), selenium (*P* < 0.001), vitamin B_6_ (*P* = 0.02), dietary fiber (*P* = 0.02), refined grains (*P* < 0.001), hydrogenated oils (*P* = 0.002) and caffeine (*P* = 0.05) in comparison to individuals in the first tertile. Consumption of other nutrients were not significantly different across tertiles of rice consumption.

**Table 2:** Dietary and nutrient intake of study participants across tertiles of rice consumption.

	Groups				*P*	Tertiles of rice consumption			
	Cases (n = 128)		Controls (n = 256)			T_1_ (< 181 g/day) (n = 129)	T_2_ (181 to < 279 g/day) (n = 121)	T_3_ (≥ 279 g/day) (n = 134)	P*
Energy (kcal/day)	2580 ± 560		2561 ± 722		0.79	2365.89 ± 705.98	2472.2 ± 513.36	2848.17 ± 674.11	< 0.001
**Nutrients**									
Proteins (g/day)	98	22	97	30	0.70	91.9 ± 33.53	93.75 ± 18.13	106.14 ± 25.4	< 0.001
Fats (g/day)	62	19	66	22	0.05	62.72 ± 20.02	64.15 ± 18.73	67.04 ± 22.97	0.22
Saturated fats (g/day)	19	7	21	9	0.08	19.41 ± 7.03	20.39 ± 8.61	21 ± 9.75	0.33
Carbohydrate (g/day)						376.52 ± 127.05	398.31 ± 97.13	470.79 ± 112.04	< 0.001
Potassium (mg/day)	4074	783	6363	1423	0.03	4208.32 ± 1491.47	4130.33 ± 1155.75	4443.24 ± 1051.8	0.12
Calcium (mg/day)	1019	263	1139	358	0.001	1090.83 ± 389.06	1065.51 ± 272.73	1135.53 ± 323.76	0.25
Selenium (mcg/day)	0.06	0.04	0.08	0.36	0.02	0.06 ± 0.04	0.06 ± 0.03	0.08 ± 0.04	< 0.001
Vitamin E (mg/day)	5	2	6	3	0.03	5.43 ± 2.65	5.39 ± 2.71	5.64 ± 3.15	0.75
Vitamin B6 (mg/day)	2	0.54	2	0.76	0.13	1.88 ± 0.83	1.85 ± 0.53	2.07 ± 0.68	0.02
Folate (mcg/day)	349	90	382	302	0.23	385.37 ± 405.57	336.61 ± 71.17	386.79 ± 129.42	0.22
Vitamin C (mg/day)	126	59	143	113	0.11	134.88 ± 69.27	124.8 ± 34.98	150.65 ± 147.77	0.12
Dietary Fiber (g/day)	23	11	23	15	0.82	22.51 ± 16.43	20.93 ± 8.63	25.69 ± 12.87	0.02
**Food groups**									
Refined grains (g/day)	501	175	421	182	< 0.001	313.41 ± 122.73	409 ± 114.78	611.92 ± 156.9	< 0.001
Whole grains (g/day)	150	134	177	108	0.03	163.65 ± 112.5	166.9 ± 104.91	147.31 ± 114.54	0.31
White meats (g/day)	30	13	33	22	0.23	30.17 ± 29.08	31.77 ± 10.79	33.35 ± 15.11	0.43
Red meats (g/day)	41	28	36	20	0.03	38.26 ± 20.81	37.58 ± 19.33	37.47 ± 19.97	0.94
Fish (g/day)	9	12	9	9	0.86	9.81 ± 13.04	7.82 ± 7.86	9.72 ± 9.04	0.22
Fruits (g/day)	325	99	361	124	0.005	360.31 ± 115.56	341.32 ± 105.05	345.09 ± 99.73	0.32
Vegetables (g/day)	258	83	274	86	0.07	274.49 ± 82.43	276.19 ± 82.93	256.44 ± 69.88	0.08
Dairy products (g/day)	309	117	355	131	0.001	350.04 ± 127.88	347.78 ± 105.7	322.62 ± 129.44	0.13
Legumes and nuts (g/day)	40	23	46	20	0.01	47.18 ± 18.64	43.45 ± 21.58	41.92 ± 18.42	0.08
Sugar-sweetened beverages (g/day)	79	67	83	74	0.57	80.96 ± 67.004	79.31 ± 58.63	85.51 ± 75.74	0.75
Partially hydrogenated vegetable oils (g/day)	15	15	9	12	< 0.001	7.73 ± 11.38	11.58 ± 13.93	13.69 ± 14.9	0.002
Non-hydrogenated vegetable oils (g/day)	7	6	8	5	0.03	8.42 ± 5.34	7.95 ± 5.58	7.26 ± 4.88	0.20
Caffeine						906 ± 431	814 ± 300	919 ± 335	0.05

Data are presented as mean ± SD or percent.

*Obtained from analysis of variance.

**Figure 2:**
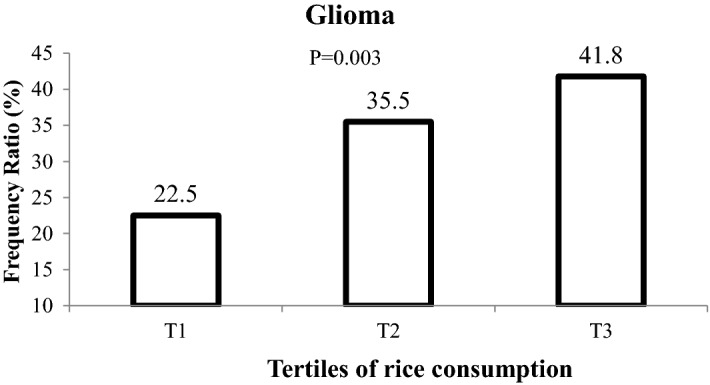
Frequency ratio of glioma across tertiles of rice consumption.

Frequency ratio of glioma across tertiles of rice consumption is shown in Fig. 2. Participants in the last category of rice consumption had significantly higher frequency of glioma compared with those in the first category (41.8 vs. 22.5%; *P* < 0.001). Multivariable-adjusted odds ratio and 95% confidence intervals for glioma across tertiles of rice consumption are shown in Table 3. Before adjustment for potential confounder factors, participants in the highest tertile of rice consumption compared with the lowest tertile, had 2.47 times greater chance for having glioma (OR: 2.47, 95% CI 1.44–4.23). Adjustment for age, gender and energy intake improved this relationship (OR: 2.80, 95% CI 1.56–5.01). The relationship was also seen when potential confounders (including physical activity (Met-h/wk), family history of glioma, family history of other cancers, education, smoking, marital status, high risk jobs, high risk living area, duration of cell phone use, history of exposure to the radiographic x-ray, history of head trauma, history of allergy, history of hypertension, supplement use, exposure to chemicals, drug use, personal hair dye use, frequency of fried food intake, frequency of barbecue/canned foods/microwave use) and dietary intakes were taking into account (OR: 2.47, 95% CI 1.03–5.92). Additional adjustment for BMI in the last model had shown that the relationship between rice consumption and glioma is independent of obesity; such that individuals in the top tertile of rice consumption had 2.46 times greater odds of glioma compared with those in the bottom tertile (OR: 2.46, 95% CI 1.01–5.97). The regression models were not disturbed by multicolinearity. The Hosmer–Lemeshow test showed goodness-of-fit of this regression model (*P* = 0.85). Multivariable-adjusted odds ratio and 95% confidence intervals for glioma across all variables included in fully-adjusted model are presented in Table 4.

**Table 3:** Multivariable-adjusted odds ratios and 95% CIs for glioma across tertiles of rice consumption.

	Tertiles of rice consumption			
	T_1_ (< 181 g/day) (n = 129)	T_2_ (181 to < 279 g/day) (n = 121)	T_3_ (≥ 279 g/day) (n = 134)	P-trend
Crude	1	1.90 (1.09–3.31)	2.47 (1.44–4.23)	0.001
Model 1^a^	1	1.93 (1.10–3.38)	2.80 (1.56–5.01)	0.001
Model 2^b^	1	1.61 (0.83–3.14)	2.25 (1.11–4.55)	0.22
Model 3^c^	1	1.38 (0.65–2.94)	2.47 (1.03–5.92)	0.04
Model 4^d^	1	1.38 (0.64–2.95)	2.46 (1.01–5.97)	0.04

^a^Model 1: adjusted for age, gender and energy intake.

^b^Model 2: further adjustments were done for physical activity, family history of cancer, family history of glioma, marital status, education, high risk occupation, high risk residential area, duration of cell phone use, supplement use, History of exposure to the radiographic x-ray, history of head trauma, history of allergy, history of hypertension, smoking, exposure to chemicals, drug use, personal hair dye use, frequent fried food intake, frequent use of barbecue, canned foods and microwave.

^c^Model 3: further adjusted for dietary intake of whole grains, red and processed meats, fruits, vegetables, white meats, legumes and nuts, and dietary fat, refined grain (except for rice) and caffeine.

^d^Model 4: additionally adjusted for BMI.

**Table 4:** Multivariable-adjusted odds ratios and 95% CIs for glioma across all studied variables.^a^

	OR (95%CI)
Rice consumption (T_3_ vs. T_1_)	2.46 (1.01–5.97)
Age (y)	0.99 (0.97–1.02)
Sex (Women vs. men)	2.45 (0.97–6.16)
Body mass index (kg/m^2^)	1.01 (0.92–1.09)
Energy intake (kcal/day)	1.00 (0.99–1.01)
Marital status (married vs. single)	2.26 (0.83–6.18)
University graduated (yes vs. no)	1.07 (0.37–3.05)
High-risk jobs^a^ (farmer vs. non-farmer)	5.48 (1.03- 29.24)
High-risk residential area (yes vs. no)	1.36 (0.65–2.84)
Duration of cell phone use (y)	0.94 (0.82–1.07)
Exposure to x-ray (yes vs. no)	1.26 (0.50–3.19)
History of head trauma (yes vs. no)	3.19 (1.61–6.35)
Allergy (yes vs. no)	0.68 (0.33–1.41)
Hypertension (yes vs. no)	0.42 (0.07–2.53)
Smoking (current smokers vs. non-smokers)	0.07 (0.02–0.22)
Frequent fried food intake (yes vs. no)	1.73 (0.71–4.22)
Frequent use of barbecue (yes vs. no)	0.95 (0.37–2.43)
Frequent microwave use (yes vs. no)	0.51 (0.19–1.34)
Frequent canned foods intake (yes vs. no)	1.50 (0.29–7.86)
Drug usage (yes vs. no)	3.36 (0.73–15.40)
Personal hair dye use (yes vs. no)	0.30 (0.12–0.73)
Exposure to chemicals (yes vs. no)	1.84 (0.72–4.73)
Family history of glioma (yes vs. no)	8.40 (3.05–23.10)
Family history of cancer (yes vs. no)	0.75 (0.39–1.44)
Supplement use (yes vs. no)	0.43 (0.15–1.28)
Physical activity (Met-h/wk)	1.01 (0.95–1.07)
Whole grain intake (g/day)	1.01 (1.00–1.01)
Red and processed meats intake (g/day)	1.04 (1.02–1.06)
Fruits intake (g/day)	0.99 (0.99–0.99)
Vegetables intake (g/day)	0.99 (0.99–1.00)
White meats intake (g/day)	0.99 (0.97–1.02)
Legumes and nuts intake (g/day)	0.98 (0.96–0.99)
Dietary fat intake (g/day)	1.00 (0.97–1.03)
Refined grain intake (except rice) (g/day)	1.01 (1.00–1.01)
Caffeine intake (g/day)	1.00 (0.99–1.00)

Adjusted for all other variables in the table.

## Discussion

We found a significant positive association between rice consumption and glioma. This association was independent of several potential confounders and obesity. To our knowledge, this is the first study investigating the relationship between rice consumption and glioma in adults.

Glioma is the most prevalent type of adult brain tumor with high malignancy. Several factors might affect its pathophysiology, including genetic, socio-cultural factors and diet^22–24^. The present study suggested that rice consumption could be a modifiable risk factor for glioma.

We found a significant positive association between rice consumption and glioma. In line with our finding, several other studies found a positive relationship between rice consumption and cancers. In a prospective study, Chyuo et al. reported a significant relation between high rice consumption and upper aerodigestive tract cancers^16^. Falk et al. have also reported a positive dose–response effect of rice intake on pancreatic cancer^17^. Some researches that investigated carbohydrate intake in relation to different cancers have reported no significant associations^14,15^. In a study among US adults, long-term consumption of total rice, white rice or brown rice was not associated with risk of developing cancer^15^. Furthermore, findings from a meta-analysis, done on observational studies, revealed no association between dietary carbohydrate intake and colorectal cancer risk^14^. However, a significant positive association was found among men. Most included studies in this meta-analysis came from Western countries and only two of the included studies with a small sample size were from Asian societies^14^. Our previous investigation on Iranian population showed that more consumption of refined grain [including the sum of Iranian white breads (Lavash, Taftoon, and Baguette), pasta, rice, boiled and fried potato, sweets and cookies] was associated with higher odds of glioma^32^. Among refined grains, the amount of rice or rice products consumed by Iranian population is more than those eaten by other nations^25,26^. Our current investigation showed that white rice consumption independently of other refined grains could increase the odds of glioma in Iranians. Low fiber content of white rice in Iranian diet along with its low content of magnesium and other essential nutrients and high amount of lead, arsenic and cadmium in rice varieties, which Iranians consume, might contribute to its relationship with glioma^38,39^.

**Figure 3:**
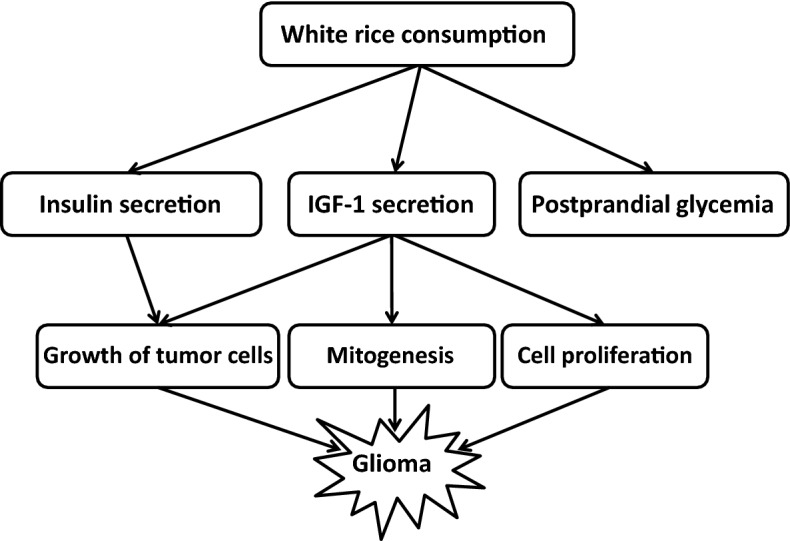
High level of white rice consumption in relation to glioma: putative underlying mechanisms.

White rice consumption as a source of refined grains affects insulin secretion and postprandial glycemia^27,28^ that are involved in the etiology of many chronic diseases, particularly cancers and glioma, as shown in Fig. 3. Elevated levels of insulin are associated with worse prognosis of breast cancer^40^, and increased insulin-like growth factor-1 (IGF-1) levels are linked to greater risk of colon, prostate and breast cancers^41–43^. These observations suggested that insulin and IGF-1 can not only modulate glucose metabolism of healthy tissues but they can also act as growth factors for tumor cells. IGF-I is an important factor in normal brain development^29^ and was documented to be over-expressed in glioma. In vitro, IGF-I receptor promote mitogenesis and differentiation in glial cells and neural cells^44^. High concentrations of IGF-I might be positively associated with risk of low-grade glioma^30^. Previous investigations have shown that tumor rates are higher in diabetic patients treated with insulin-releasing drugs, but not in patients treated with metformin, which did not increase insulin levels^29^. High blood glucose levels are associated with worse prognosis in patients with glioblastoma^45^. Therefore, reducing glucose availability by restricted refined carbohydrates intake might affect tumor growth.

The present study has some strength and weaknesses. We evaluated the linkage between rice consumption and glioma in adults for the first time. A wide range of potential covariates was taken into account in the analysis to have an independent relation between rice consumption and odds of glioma. Furthermore, only newly-diagnosed patients with glioma cases were enrolled in the study in order to reduce the possibility of changing usual dietary intakes in these patients. It must also be kept in mind that the study comes from the Middle East where rice consumption is high. Some limitations are also needed to be taken into account while interpreting the findings. Due to case–control design, the study was subject to some biases including selection bias and recall bias. We enrolled newly-diagnosed cases in this study to reduce the recall bias. However, since dietary assessment occurred after diagnosis of the disease, cases might recall their past diet differently in the context of their cancer diagnosis. Cases might also have altered their dietary intakes before diagnosis due to early symptoms of the disease. Owing to such design of the study, we could not confer causality. We used a validated semi-quantitative food frequency questionnaire (FFQ) to assess dietary intakes of participants which was self-reported; this might lead to misclassification of participants. In addition, our applied FFQ was assessed dietary intakes of the preceding year, while cancer development might require a long time exposure to diet. Glioma was defined based on ICD-O-2 morphology; the results might be different if updated classifications (such as ICD-10 and ICD-11) would be applied for disease diagnosis. Participation rate was lower for control group than for case group, which might result in selection bias. In addition, although we controlled for several confounders, one cannot exclude the possibility of residual confounding. Cases and controls were enrolled at the same period of time and from the same place of residence. However, slightly different food availability, dietary preference or dietary custom of the study population might lead to selection bias; therefore, some caution should be applied in extrapolation of our findings to general population. We could not perform stratified analyses base on glioma subtypes, because the type of tumor was not determined for all cases. Finally, as the dietary intakes of Middle Eastern population are different from Western nations, the generalizability of the findings to other populations should be made with cautious.

In conclusion, we found that rice consumption was positively associated with risk of glioma in Iranian adults. Further studies, particularly with prospective design, are required to confirm this finding.

## Abbreviations

CNS: Central nervous system
IPAQ: International Physical Activity Questionnaire
MET-h/wk: Metabolic equivalent task-hours per week
BMI: Body mass intake
CHO: Carbohydrate
FFQs: Food Frequency Questionnaire
N4: Nutritionist 4
